# Neuroinflammation signatures in dorsal root ganglia following chronic constriction injury

**DOI:** 10.1016/j.heliyon.2024.e31481

**Published:** 2024-05-17

**Authors:** Yao Qu, Ruirui Cai, Qiao Li, Han Wang, Laijin Lu

**Affiliations:** a Department of Hand and Foot Surgery, The First Hospital of Jilin University, No.1 Xinmin Street, Changchun, 130021, Jilin, China; b Department of Pain Medicine, The First Hospital of Jilin University, Changchun, 130021, Jilin, China; c School of Information Science and Technology, Institute of Computational Biology, Northeast Normal University, No.2555 Jingyue Street, Changchun, 130117, Jilin, China; d Department of Spinal Surgery, The First Hospital of Jilin University, Changchun, 130021, Jilin, China

**Keywords:** Neuropathic pain, Dorsal root ganglia, Chronic constriction injury, RNA sequencing, Transcriptome, Comprehensive analysis

## Abstract

Neuropathic pain (NP) is a common debilitating chronic pain condition with limited effective therapeutics. Further investigating mechanisms underlying NP is therefore of great importance for discovering more promising therapeutic targets. In the current study, we employed high-throughput RNA sequencing to explore transcriptome profiles of mRNAs and microRNAs in the dorsal root ganglia (DRG) following chronic constriction injury (CCI) and also integrated published datasets for comprehensive analysis. First, we established CCI rat model confirmed by behavioral testings, and excavated 467 differentially expressed mRNAs (DEGs) and 16 differentially expressed microRNAs (DEmiRNAs) in the ipsilateral lumbar 4–6 DRG of CCI rats 11 days after surgery. Functional enrichment analysis of 337 upregulated DEGs showed that most of the DEGs were enriched in inflammation- and immune-associated biological processes and signaling pathways. The protein-protein interaction networks were constructed and hub DEGs were screened. Besides hub DEGs, we also identified 113 overlapped DEGs by intersecting our dataset with dataset GSE100122. Subsequently, we predicted potential miRNA-mRNA regulatory pairs using DEmiRNAs and a given set of key DEGs (including hub and overlapped DEGs). By integrative analysis, we found commonly differentially expressed mRNAs and miRNAs following CCI of different time points and different nerve injury types. Highlighted mRNAs include Atf3, Vip, Gal, Npy, Adcyap1, Reg3b, Jun, Cd74, Gadd45a, Tgm1, Csrp3, Sprr1a, Serpina3n, Gap43, Serpinb2 and Vtcn1, while miRNAs include miR-21-5p, miR-34a-5p, miR-200a-3p, miR-130a-5p, miR-216b-5p, miR-217-5p, and miR-541-5p. Additionally, 15 DEGs, including macrophages-specific (Cx3cr1, Arg1, Cd68, Csf1r) and the ones related to macrophages’ involvement in NP (Ccl2, Fcgr3a, Bdnf, Ctss, Tyrobp) were verified by qRT-PCR. By functional experiments in future studies, promising therapeutic targets for NP treatment may be identified among these mRNAs and miRNAs.

## 1 Introduction

The Neuropathic pain (NP), caused by a lesion or disease of the somatosensory nervous system itself, is a debilitating chronic pain condition with an estimated prevalence of 6.9%–10 % in the general population. This common clinical condition severely impairs the life quality of patients and causes a large healthcare burden [1,2]. Unfortunately, currently available analgesics fail to provide sufficient pain relief for patients suffering from NP due to the complicated mechanisms of NP [3,4]. Further efforts are therefore urgently required to gain insights into the mechanisms underlying NP for more effective treatments.

The dorsal root ganglia (DRG) are complex tissue containing cell bodies of sensory neurons, satallite glial cells, schwann cells and resident immune cells, etc. DRG are located between the spinal cord dorsal horn and peripheral nerve terminals along the somatosensory nociceptive pathway responsible for pain transmitting and modulating, of which DRG is the peripheral part while the spinal cord and super spinal regions are the central counterparts. Unlike central regions of the nociceptive pathway, DRG anatomically lack a blood-nerve barrier [5], making it an ideal target for analgesics. A better understanding of how pathogenic factors-induced cellular and molecular changes of DRG contribute to the genesis and persistence of NP is therefore of great importance.

Using microarray or RNA sequencing (RNA-seq), preclinical studies have investigated transcriptome profiles of mRNA and non-coding RNA in the DRG of different peripheral nerve injury (PNI) induced-NP models, including widely used sciatic nerve chronic constriction injury (CCI), spared nerve injury, spinal nerve ligation, partial sciatic nerve ligation, etc. [[6], [7], [8], [9], [10], [11], [12], [13], [14]]. Studies focusing on DRG transcriptome in the context of CCI-induced NP were scarce. Stephens et al. profiled global mRNA changes in DRG 14 days after CCI [9]. A very recent study from Guo et al. revealed expression profiles of microRNA (miRNAs) and mRNAs in DRG at 28 days after CCI, a late maintenance phase of NP in preclinical settings [13].

In the present study, we established the CCI rat model and employed RNA-seq to investigate transcriptome signatures in bulk DRG 11 days post-surgery. By utilizing bioinformatic tools and integrating previous high-throughput datasets, we provide an integrative insight into the molecular mechanisms underlying maintenance phase of NP in response to CCI. The highlighted mRNAs and miRNAs may serve as promising therapeutic targets for future NP treatment.

## 2 Results

### 2.1 Pain-like behavioral signs induced by CCI surgery

**Fig. 1:**
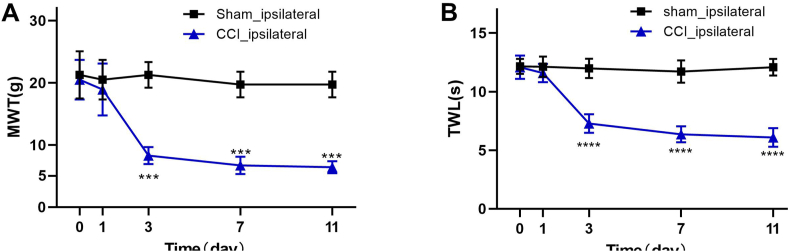
Behavioral testings at different time points. (A) Mechanical withdraw threshold (MWT) and (B) Thermal withdraw latency (TWL) of ipsilateral hind paw of chronic constriction injury (CCI) and sham group rats at different time points. Values are expressed as mean ± SD. ***p ≤ 0.001, ****p ≤ 0.0001, CCI group (n = 7) vs baseline and sham group (n = 7) at each time point.

MWT and TWL of rats were evaluated and quantified by von Frey filaments and Plantar Analgesia Tester respectively at different time points. The CCI group (n = 7) exhibited a significant decrease in MWT and TWL on the ipsilateral hind paw 3, 7, and 11 days after surgery compared to the baseline and the sham group (n = 7), indicating the CCI model was successfully established in these rats which developed pain-like behaviors after nerve injury, including mechanical allodynia and thermal hypersensitivity, while rats in the sham group showed no significant changes of MWT and TWL after surgery (Fig. 1A and B). Three rats in each group were randomly chosen for RNA-seq analysis.

### 2.2 Differentially expressed mRNAs and miRNAs identified by RNA-seq

**Fig. 2:**
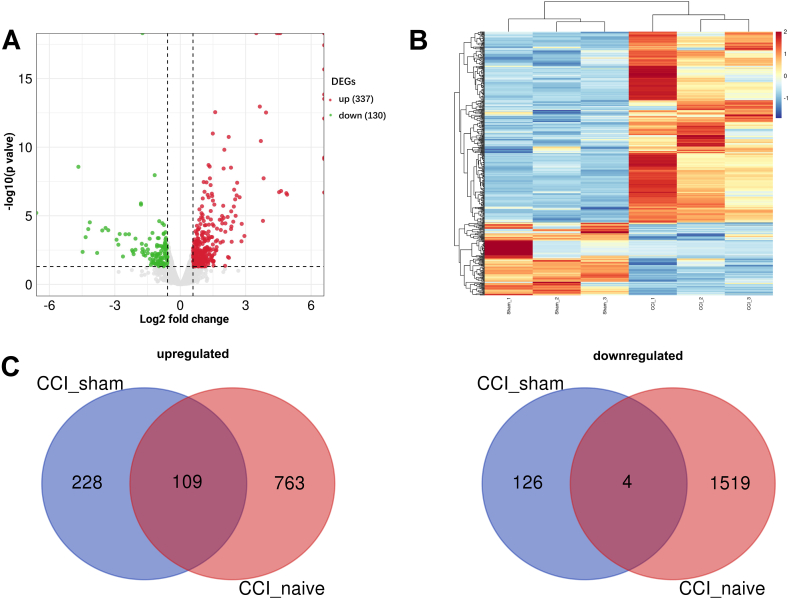
Differentially expressed mRNAs (DEGs) in rat ipsilateral DRGs 11 days after chronic constriction injury. (A) Volcano plot showing upregulated and downregulated DEGs. Red dots and green dots indicate genes with significantly upregulated and downregulated expression, respectively, while gray dots indicate genes with no significantly difference. (B) Heatmap showing hierarchical clustering of DEGs. Upregulated and downregulated genes are colored in red and blue, respectively. (C) Venn diagrams present the number of upregulated DEGs (left) and downregulated DEGs (right) that are unique or shared between our dataset (CCI_sham, blue circle) and GSE100122 dataset (CCI_naïve, red circle).

We employed RNA-seq to detect transcriptomic profiles of ipsilateral L4–6 DRG from CCI rats. Following the filtering criteria of p < 0.05, and |log2 fold change| ≥0.58, we identified 467 significantly differentially expressed mRNAs (DEGs) (337 upregulated and 130 downregulated) in the CCI group 11 days after surgery compared to the sham group. Volcano plot and heatmap exhibiting the overall distribution of upregulated and downregulated DEGs are shown in Fig. 2A and B. Furthermore, following the same processing procedures, 2394 DEGs were screened from GSE100122 raw data. Excluding DEGs with inconsistent regulation, we intersected these two DEGs datasets and found 109 overlapped upregulated DEGs and 4 downregulated DEGs, as shown in the venn diagrams (Fig. 2C). On the other hand, a total of 16 significantly differentially expressed miRNAs (DEmiRNAs) were identified, with detailed information listed in Table 1.

**Table 1:** Differentially expressed miRNAs of CCI group compared with sham group.

miRNA_name	FC	Log2 FC	P value	Regulation	Predicted target genes^※^
miR-21-5p	1.87	0.90	2.06E-04	up	–
miR-216a-5p	0.32	−1.65	5.55E-04	down	Tdrd12: ENSRNOT00000017021
PC-3p-8573_136	4.13	2.04	3.25E-03	up	–
miR-217-5p	0.47	−1.08	3.70E-03	down	Tph2: ENSRNOT00000005157 Vip: ENSRNOT00000025477 Vtcn1: ENSRNOT00000020566
miR-376b-5p	0.82	−0.28	4.68E-03	down	–
miR-200a-3p	0.47	−1.07	6.94E-03	down	Atf3: ENSRNOT00000005085 Serpinb2: ENSRNOT00000003409
miR-146b-3p	0.63	−0.67	1.41E-02	down	Vtcn1: ENSRNOT00000020566
miR-34a-5p	0.76	−0.39	1.47E-02	down	Cldn4: ENSRNOT00000002003 Gal: ENSRNOT00000020425 Ptpn5: ENSRNOT00000018860 Smagp: ENSRNOT00000073536 Stac2: ENSRNOT00000091978 Tdrd12: ENSRNOT00000017021 Vtcn1: ENSRNOT00000020566
miR-216b-5p	0.32	−1.63	1.63E-02	down	Sox11: ENSRNOT00000045963 Vtcn1: ENSRNOT00000020566
miR-34b-3p	0.74	−0.43	2.29E-02	down	–
miR-15a-5p	0.14	−2.84	2.63E-02	down	–
miR-130a-5p	1.66	0.73	2.68E-02	up	Rasgrp3: ENSRNOT00000051239
miR-541-5p	1.34	0.42	2.78E-02	up	Gabra5: ENSRNOT00000093306 Mapk7: ENSRNOT00000057864
miR-1188-5p	1.59	0.67	4.11E-02	up	Mapk7: ENSRNOT00000057864
miR-98-3p	0.45	−1.15	4.82E-02	down	–
PC-3p-8170_145376	2.05	1.04	4.89E-02	up	–

Names of miRNAs beginning with PC (Predicted Candidate) are brand new miRNAs. FC: fold change. * The predicted target genes come from 113 overlapped genes and 58 hub genes of 5 PPI modules, name of each gene is followed by transcript ID. - means no corresponding predicted target genes.

### 2.3 Functional enrichment analysis of DEGs reveal neuroimmune signatures

**Fig. 3:**
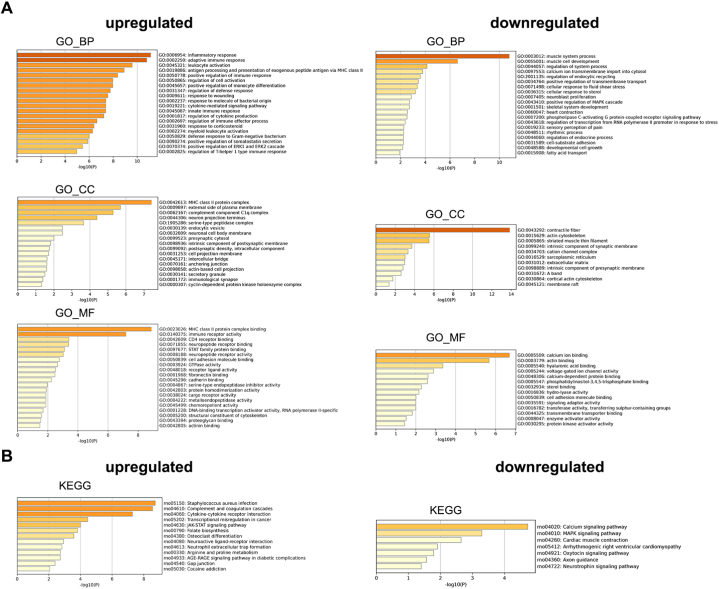
Functional enrichment analysis of DEGs. (A) GO terms significantly enriched by upregulated (left) and downregulated (right) DEGs. GO: Gene Ontology; BP: biological process; CC: cellular component; MF: molecular function. (B) KEGG pathways significantly enriched by upregulated (left) and downregulated (right) DEGs. KEGG: Kyoto Encyclopedia of Genes and Genomes. The horizontal axis represents the value of -log10 (p-value), the vertical axis represents GO terms or KEGG pathway names. Colors represent p-values, deeper color indicate smaller p-value.

To further understand the functions of DEGs, we carried out GO analysis to reveal significantly enriched biological process (BP), cellular component (CC), and molecular function (MF). For the upregulated DEGs, the enriched biological processes included inflammatory response, adaptive immune response, leukocyte activation, antigen processing and presentation of exogenous peptide antigen via MHC class Ⅱ, positive regulation of monocyte differentiation, cytokine-mediated signaling pathway, innate immune response, etc. Enriched molecular functions were MHC class Ⅱ protein complex binding, immune receptor activity, CD4 receptor binding, neuropeptide receptor binding, etc. Cellular components were enriched in MHC class Ⅱ protein complex, external side of plasma membrane, complement component C1q complex, neuron projection terminus, serine-type peptidase complex, etc. Corresponding GO analysis results of the downregulated DEGs are also presented (Fig. 3A). KEGG analysis identified that the upregulated DEGs were primarily enriched in the following pathways: staphylococcus aureus infection, complement and coagulation cascades, cytokine-cytokine receptor interaction, JAK-STAT signaling pathway, neuroactive ligand-receptor interaction, etc., while the downregulated DEGs were enriched in calcium signaling pathway, MAPK signaling pathway, axon guidance, neurotrophin signaling pathway, etc (Fig. 3B). Enrichment analysis of the upregulated DEGs indicated that intense inflammatory and immune responses occurred within the affected DRGs following CCI.

### 2.4 PPI network of identified DEGs

**Fig. 4:**
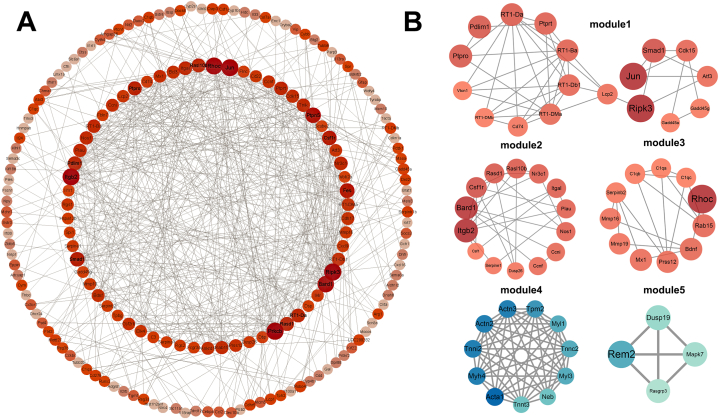
Protein-protein interaction (PPI) network and modules of DEGs. (A) PPI network of 337 upregulated DEGs. (B) 5 significant modules extracted from PPI network by MCODE. Module 1–3 with gradient red color are derived from upregulated DEGs, while module 4–5 with gradient blue color are from downregulated DEGs. The size and color of the nodes indicate the connection degree of genes, larger size along with deeper color indicate higher degree.

To identify potential interactions between protein-encoding DEGs, we constructed the PPI network by Metascape and visualized it by Cytoscape. Here we only present the PPI network of 337 upregulated DEGs because they are of particular research interest. Including genes with at least one connection with other genes and excluding 2 small networks with 5 genes isolated from the big one, a network consisting of 180 interconnecting nodes (genes) linked by 526 edges was constructed (Fig. 4A). Further, we extracted 3 significant PPI modules of upregulated DEGs and 2 significant modules of downregulated DEGs from the PPI networks by MCODE (Fig. 4B). 58 hub DEGs within these 5 PPI modules were considered as key genes for further analysis.

### 2.5 Regulatory analysis of mRNAs-miRNAs pairs

For miRNA-mRNA regulation analysis, we limited the candidate mRNAs of interest to 113 overlapped DEGs and 58 hub DEGs of 5 PPI modules. A total of 155 key mRNAs, with 16 mRNAs shared by these two DEGs sets were selected (Supplementary Fig. S1A). Following the prediction intensity of Targetscan and miRanda, we obtained highly predicted miRNA-miRNA regulatory pairs. Among the target genes predicted, three genes, namely Atf3, Serpinb2, and Vtcn1, were shared by overlapped DEGs and hub DEGs. Atf3 and Serpinb2 were predictively targeted by miR-200a-3p, while Vtcn1, was by miR-34a-5p, miR-217-5p, miR-146b-3p, miR-216b-5p. Besides Vtcn1, miR-34a-5p which is known to be associated with NP was also predicted to target other 6 genes, Gal, Cldn4, Smagp, Stac2, Tdrd12, and Vtcn1. The detailed regulatory pairs are shown in Table 1, Supplementary Fig. S1B.

### 2.6 Validation of RNA-seq results by qRT-PCR

To validate the results of RNA-seq, 15 upregulated DEGs, including macrophages-specific genes (Cx3cr1, Arg1, Cd68, Csf1r), shared genes (Table 2: Gadd45a, Csrp3, Adcyap1), several known (Panx1, Sox11, Ccl2, Fcgr3a, Bdnf, Tyrobp, Ctss) and undetermined (Vtcn1) pain-related genes were selected and verified by qRT-PCR. The results showed that the expression of all these genes in the DRG from CCI group compared with sham group were significantly upregulated (Supplementary Fig. S2), which was consistent with the RNA-seq results. The result of qRT-PCR provided evidence that the RNA-seq dataset we obtained was reliable.

**Table 2:** Summary of shared DEGs between four studies.

Comparison with	Shared DEGs
Stephens et al., 2019 [9]	See Fig. S1A and 113 genes in the red circle
Stephens et al., 2019 [15] and Guo et al., 2021 [13] a	Atf3, Vip, Gal, Npy, Adcyap1, Reg3b, Cacna2d1, Nos1, Gch1, Gabra5, Stac2, Pde6b
Sun et al., 2020 [11]	Atf3, Vip, Gal, Npy, Adcyap1, Reg3b, Jun, Cd74, Gadd45a, Tgm1, Csrp3, Sprr1a, Serpina3n, Gap43
Stephens et al., 2019 [9] and Sun et al., 2020 [11]	Atf3, Vip, Gal, Npy, Adcyap1, Reg3b, Jun, Gadd45a, Tgm1, Csrp3, Sprr1a, Serpina3n, Gap43

a Only compare with limited DEGs that were exhibited in the literature.

## 3 Discussion

Cellular and molecular changes in response to peripheral nerve insults within DRG can either be adaptive by promoting axon regeneration or maladaptive by enhancing DRG neuronal excitability and thus pain hypersensitivity. High-throughput RNA sequencing enables fully revealing changes of gene expression caused by peripheral nerve injury at the transcriptional level and discovering mechanisms underlying NP.

As shown in the results, significantly enriched GO and KEGG terms of upregulated DEGs strongly indicated that inflammatory response was the major event within the affected lumbar DRGs 11 days after CCI surgery, with considerable DEGs participating. Among these DEGs, we identified dozens of upregulated genes known to be exclusively expressed by macrophages [[16], [17], [18]], and verified expression of some representative ones (Csf1r, Cx3cr1, Cd68, Arg1) by qRT-PCR. Upregulation of these genes could be explained by previous peripheral nerve injury studies, which have detected increased macrophages number in the injured DRG (or trigeminal ganglia) beginning from as early as 3 days to at least 4 weeks by immunohistology [19,20], fluorescence-activated cell sorting (FACS) [15,16], or single-cell RNA sequencing (scRNA-seq) (Avraham et al., 2021) and single-nucleus RNA sequencing (snRNA-seq) techniques [17]. Hub gene Csf1 within module 2, is a cytokine that has been demonstrated the most critical contributor to macrophages expansion in DRG after peripheral nerve injury by acting via Csf1 receptor (Csf1r, within module 2) expressed on macrophages [15,21]. The Csf1-Csf1r interaction, both of which are hub genes of module 2, further rationalize the above deduction; however, whether the expansion result from a proliferation of resident macrophages and/or recruitment and infiltration of myeloid macrophages into the DRG remains controversial, which could not get a clue herein. Besides, we could not tell the activated macrophages were dominant by pro-inflammatory M1 type or anti-inflammatory type M2 macrophages, merely from upregulation of Arg1, an M2 macrophages marker, and no significant upregulation of Nos2, which encode iNOS and mark activated M1 macrophages. Based on the above, our transcriptome data implicate those macrophages, by expansion and upregulating functional molecules, play a pivotal role in the neuroimmune reaction in the context of CCI-induced NP at a relatively early maintenance phase. Our findings were supported by previous studies which have provided evidence that, following peripheral nerve injury, DRG macrophages are critical contributors to both the initiation and maintenance of NP in rodents, through communication with nociceptive neurons [15,21,22]. Interestingly, Relative to the GO biological process term ‘innate immune response’ implying the involvement of macrophages, the terms ‘adaptive immune response’ and ‘regulation of T-helper 1 type immune response’ otherwise highly implicated the involvement of T cells. Besides, BP term ‘antigen processing and presentation of exogenous peptide antigen via MHC class Ⅱ’ suggest the biological process of interaction between macrophages and T cells. Previous studies have provided evidence as to the deduction, such as CD4^+^ T cells were recruited into injured DRG after CCI [23], depletion of macrophages or T cells reduced neuropathic tactile allodynia [24], etc. Together, we suggest both macrophages and T cells, are activated in response to CCI at 11 days post-surgery, and play central roles among immune cells, with the former dominant, in the injury-induced neuroimmune events within DRG by interacting with other types of cells and contributing to the persistence of NP.

In the context of sciatic nerve CCI, to explore key genes in injured DRG contributing to the persistence of NP, we compared our dataset with another RNA-seq dataset from a similar study [9] and identified 109 upregulated and 4 downregulated DEGs in common. Screening out both from raw data following the same procedures, the distinct number and profiles of DEGs in these two studies are probably due to the difference of the control group. Stephens et al. use naïve rats as the control group, and thus may presumedly capture gene expression changes not only associated with nerve constriction injury but also with skin incision, nerve terminal damage, and inflammatory response of deep muscle tissue. Besides, the two studies took 11 days and 14 days post-CCI as endpoints respectively, both of which were considered to be in the maintenance phase of NP [14]; however, the fact that transcriptional changes are dynamic in nature might also help to explain the distinction. Furthermore, a different number of biological replicates, heterogeneity between studies with respect to high-throughput platform, ligature, and tightness of ligation, etc., might also contribute to the discrepancies. For further integrative analysis, we also referred to partial data shown by Guo et al. [13], and found that at least Atf3, Vip, Gal, Npy, Adcyap1, Reg3b, Cacna2d1, Nos1, Gch1, Gabra5, Stac2, Pde6b are consistently upregulated across 11-14-28 day after CCI, that is to say from a relatively early to late maintenance stage of NP in the case of a rat model. Additionally, our dataset shared 14 out of 18 DEGs on the TOP 100 list with one existing overlapped data, which combined ipsilateral DRG transcriptional datasets of the other three peripheral nerve injury NP models from different studies [11]. Among the TOP 100 DEGs of each dataset, the DEGs found to be consistently upregulated were Npy, Gal, Atf3, Reg3b, Vip, Tgm1, Csrp3, Sprr1a, Cd74, Gadd45a, Jun, Adcyap1, Serpina3n, Gap43, 13 of which were also shared with our 113 overlapped DEGs except Cd74. Thereinto, Atf3, Jun, Cd74 and Gadd45a were hub genes of PPI module1 (Fig. 4B). Though all these genes are commonly injury-induced, Atf3, a transcription factor, which is induced rapidly after nerve injury, likely play the central role among them. By acting with other injury-induced transcription factors upregulated in our study, such as Jun, Atf3 could positively regulate the expression of Npy, Gal, Vip, Sprr1a, Adcyap1, Gap43, etc., all of which play vital roles both in NP and axon regeneration [17,[25], [26], [27]]. Notably, by integrating these high-throughput datasets, we found considerable similarity in transcriptional signatures regardless of the same CCI model with different time points or different NP models induced by various peripheral nerve injuries. These genes are likely involved in the common mechanisms of NP and might serve as therapeutic targets. (Table 2 summarizes shared DEGs of our dataset and the other three datasets discussed above).

MicroRNA (miRNA) is a small non-coding RNA that can negatively regulate multiple gene expressions at the post-transcriptional level by binding with the 3′-UTR of mRNAs. MiRNAs are involved in a variety of diseases by regulating disease-related mRNAs, including neuropathic pain [28]. However, the roles of miRNAs remain inadequately explored in the context of NP. Among DEmiRNAs in our study, miR-21-5p was the most significantly expressed, which has been consistently reported to upregulated in the DRG neurons of NP rodents after various peripheral nerve injuries [7,22,[28], [29], [30]], deletion or overexpression of miR-21-5p could attenuate or enhance pain hypersensitivity, respectively [22,30]. Simeoli et al. provided an interesting suggestion that injury-induced miR-21-5p drives pain hypersensitivity probably through mediating neuron-macrophages communications. Specifically, miR-21-5p is released as part of exosomal cargo from neurons and phagocytosed by macrophages, which then polarize toward a pro-inflammatory over an anti-inflammatory phenotype. Pro-inflammatory macrophages in turn release inflammatory mediators to sensitize nociceptive neurons, leading to sensory hypersensitivity eventually [22]. Though the importance of miR-21-5p, we didn't identify genes of interest being its target, maybe further exploration is needed in the future. As for another downregulated DEmiRNAs, miR-34a-5p, which has been also reported involved in NP [28], was predicted to target 7 upregulated mRNA transcripts in our study. No known studies have exhibited these pairings. Considering Gal, which is a well-known pain-related gene encoding neuropeptide galanin [31], we would prefer to focus on miR-34a-5p-Gal pairing for deep investigation. With similar consideration, though miR-216b-5p, miR-541-5p, miR-217-5p have no known evidence of involvement in NP, they were predicted to target known pain genes, i.e., Sox11, Gabra5, Vip, respectively [25,32,33]. Thus, miR-216b-5p-Sox11, miR-541-5p-Gabra5, miR-217-5p-Vip, are desired further investigations. MiR-130a-5p has been implicated in NP [34], while its predicted target, Rasgrp3 was otherwise. More investigations are needed on both. Actually, the DEmiRNA arousing our interest most is miR-200a-3p, of which target mRNAs, Atf3 and Serpinb2, are both on the list of 113 overlapped DEGs and hub genes simultaneously. As we have discussed above, Atf3 is a pain gene that plays a pivotal role within DRG in PNI-induced NP states and axon regeneration. Serpinb2 is known as a regulator of the inflammatory process and has been found to contribute to macrophages infiltration via enhancing Ccl2 expression in different disease models [35,36]. Besides, miR-200a-3p has been reported to modulate gene expression in comorbid pain and depression [37]. Therefore, miR-200a-3p, Atf3, Serpinb2, and their regulatory relationships should be put most emphasis on for next step investigation. Same as Atf3 and Serpinb2, Vtcn1 is the third core mRNA shared by two sets of candidate mRNAs. It was predicted to be targeted by miR-34a-5p, miR-217-5p, miR-146b-3p and miR-216b-5p. Vtcn1, also known as B7–H4, is similar to Serpinb2, it is not directly related to pain; however, its importance in immune response has made it a therapeutic target for the treatment of tumors, inflammation, autoimmune diseases, and organ transplantation [38]. Therefore, we are willing to explore its function in the context of NP in the future. Taken together, we speculate except miR-21-5p, miR-34a-5p, miR-200a-3p, miR-130a-5p which have been implicated in NP, miRNAs including miR-216b-5p, miR-217-5p, and miR-541-5p are also promising candidates for NP research because they predictively target pain-genes. Meanwhile, we identified another two interesting key genes, Serpinb2 and Vtcn1, which might present promising therapeutic targets.

There are still some limitations. The RNA-seq was carried out on bulk DRG tissue, which cannot represent the transcriptome changes in different individual cell types, especially those of research interests, such as immune cells, satellite glial cells, sensory neurons, and corresponding subtypes, etc. With the emerging scRNA-seq and snRNA-seq techniques, increasing studies tend to explore PNI-induced transcriptional alterations of DRG neurons and non-neuronal cells at the single-cell level [17,18,39,40]. For further research, we could employ this powerful technology to investigate the expression profiles of DRG subtype cells in the context of CCI-induced NP, which will contribute to drawing the whole physiological and pathological cell transcriptome atlas of all kinds of PNI-evoked NP conditions. That will be sure to provide further insight into the underlying mechanisms of NP and facilitate the development of pain therapies. Additionally, we did not include female rats in our study, sexual dimorphisms has been increasingly reported in the context of neuropathic pain [41]. In future studies, we will conduct preclinical and clinical experiments on both sexes to elucidate the roles of candidate mRNAs and miRNAs we have identified herein.

## 4 Materials and methods

### 4.1 Animals

Male Sprague-Dawley (SD) rats (6–8 weeks, weighing 180–220 g) were acquired from Charles River company (Beijing, China) and group-housed (2 per cage) under standard conditions (specific pathogen-free, temperature: 22–25 °C, humidity: 45–50 %, 12 h light/dark cycle) at the Laboratory Animal Center of the First Hospital of Jilin University, with free access to food and water. All animals were habituated to the environments for two-three weeks before experiments. The body weights of rats were 340–380 g at the time of surgery. All experimental procedures were approved by the Ethics Committee of the First Hospital of Jilin University (Approval No. 20210581) and followed the Guidelines for Care and Use of Laboratory Animals of Jilin University. All efforts were made to minimize the rat's number and their suffering.

### 4.2 Surgery

SD rats were randomly assigned to the CCI or sham group. The CCI and sham models were established as previously described [42]. Briefly, after anesthesia induction with 4 % isoflurane and maintenance with 2 % isoflurane, a rat was fixed in a prone position on the operating table, after shaving and sterilizing with iodine and 75 % ethanol, we cut the skin and subcutaneous tissue with a scalpel at the left middle thigh. Then we carefully dissected the sciatic nerve underlying the biceps femoris with scissors along the muscle fibers. About 7 mm-long nerve proximal to the sciatic trifurcation was exposed and then loosely tied by 4 knots with 4-0 absorbable suture with 1 mm spacing. The desired degree of knot tightness was to retard the circulation through the superficial epineural vasculature and sometimes produced a small, brief twitch in the muscle surrounding the exposure. For the sham group rats, the left sciatic nerve was identically exposed without ligation. At last, the incision was closed in layers with 4-0 non-absorbable sutures. Rats were monitored daily after surgery. It is also worth mentioning that the Naïve group of Stephens et al. [9] underwent no surgical procedures.

### 4.3 Behavioral testing

The rats were allowed to acclimate to the testing environment for 30 min daily beginning from 3 days before surgery, as well as acclimate for 30 min before behavioral testing at the time point of 3 h before and 1, 3, 7, and 11 days after surgery. For mechanical withdrawal threshold (MWT) testing, the rats were placed individually in plexiglass chambers on top of a wire mesh platform. The plantar surface of the hind paw in the innervation area of the sciatic nerve was stimulated with ascending force intensities of von Frey filaments (Touch Test®, North Coast Medical, CA, USA) to determine MWT. A positive response was defined as withdrawing, shaking, or licking the paw. The number of positive responses for each force was recorded. If the response rate was more than 40 % (i.e., withdrawal response was elicited in two or more out of five applications) to a given stimulus intensity, testing stopped and the force of the last von Frey filament was designated as the MWT. Significant decreases in MWT were interpreted as mechanical allodynia.

For thermal withdrawal latency (TWL) testing, the rats were placed individually in plexiglass chambers with a glass floor. The BME-410C Plantar Analgesia Tester (Institute of Biomedical Engineering, CAMS, China) was used for testing. The heat source from a light bulb was positioned underneath the rat and focused on the plantar surface of the hind paw, the time taken to withdraw from the heat stimulus was recorded as TWL. The intensity of the light source was adjusted to produce withdrawal latencies of 10–12s in naïve rats before formal testing, with a pre-determined 20s cut-off to prevent tissue damage. Significant decreases in TWL were interpreted as thermal hypersensitivity.

### 4.4 Tissue collection and RNA extraction

At 11 days post-surgery after behavioral testing, rats with expected behavioral performances were assigned to the CCI group (n = 7) and sham group (n = 7). Lumbar (L)4–6 DRG were harvested from one rat per sample. Briefly, the rats were euthanized with isoflurane (5 %) and perfused with normal saline (250 ml, 4 °C) through the ascending aorta. The ipsilateral L4–L6 DRG of the surgery side (left) were rapidly dissected in an RNase-free environment. The samples were snap-froze in liquid nitrogen and then stored at −80 °C until use. The total RNA of each sample was extracted by TRK-1002U (LC Sciences, Houston, Texas, USA) according to the manufacturer's protocol. The quality and quantity of RNA in each sample were measured by Nanodrop ND-1000 (NanoDrop, Wilmington, DE, USA). The RNA integrity number (RIN) was assessed by Agilent 2100 Bioanalyzer (Agilent Technologies, Santa Clara, CA) with RIN >7.0. Three rats of each group formed three biological replicates.

### 4.5 cDNA library preparation and RNA sequencing

Briefly, approximately 2 μg of total RNA from each sample was used to prepare the cDNA library of mRNA by ribosomal RNA depletion method using Ribo-Zero rRNA Removal Kit (Illumina, San Diego, CA, USA), while about 2 μg of total RNA was used to prepare the cDNA library of miRNA by TruSeq Small RNA Sample Prep Kits (Illumina, San Diego, USA) following the manufacturer's instructions. The quality of the final cDNA library was assessed with an Agilent 2100 Bioanalyzer (Agilent Technologies, Santa Clara, CA) for further RNA-seq. The cDNA library of mRNA was subjected to deep sequencing using an Illumina Novaseq 6000 sequencing platform with a paired-end method, while the cDNA library of miRNA was on an Illumina Hiseq 2500 sequencing platform. 12G raw data per sample were obtained on average.

### 4.6 Screening of differentially expressed mRNAs and miRNAs

After being strictly filtered and screened from raw reads, the clean reads of mRNA were mapped to the *Rattus norvegicus* reference Genome (V101) by HISAT(V2.0.4). The mapped reads of each sample were assembled using StringTie. After the final transcriptome was generated, StringTie (V1.3.0) and edgeR (https://bioconductor.org/packages/release/bioc/html/edgeR.html) were used to estimate the expression levels of all transcripts, which were then normalized with the FPKM (Fragments Per Kilobase of exon model per Million mapped reads) algorithm. Compared to sham rats, the significantly differentially expressed mRNAs (DEGs) of CCI rats were screened by edgeR using a cut-off of |log2 fold change| ≥0.58 and p < 0.05. For miRNA, the clean reads of miRNA were mapped to the reference genome in the miRBase 22.0 (http://www.mirbase.org/). Significantly differentially expressed miRNAs (DEmiRNAs) were screened using *t*-test based on normalized deep-sequencing counts with the same cut-off. The volcano plot and heatmap of DEGs were generated via the R package ggplot2 and pheatmap. Furthermore, to compare with another study similarly exploring the global mRNA profiles of rat DRG after CCI, raw data of GSE100122 RNA-seq dataset was downloaded from Gene Expression Omnibus (GEO) (https://www.ncbi.nlm.nih.gov/geo/), The DEGs were screened with the same procedures as described above. The intersection of our DEGs and DEGs derived from GSE100122 was conducted by the online tool Draw Venn Diagram (http://bioinformatics.psb.ugent.be/webtools/Venn/) for further analysis. Our mRNA sequencing data has been submitted to the GEO repository and assigned GEO accession numbers: GSE212311.

### 4.7 Functional enrichment analysis

For functional enrichment analysis of the 467 DEGs, the upregulated and downregulated DEGs were subjected to Gene Ontology (GO) and Kyoto Encyclopedia of Genes and Genomes (KEGG) pathways analysis respectively with the online Metascape database (http://metascape.org/gp/index.html#/main/step1). Metascape is a powerful tool that integrates several authoritative functional databases such as GO, KEGG, STRING, BioGrid and Uniprot, etc. for gene and protein bioinformatics analysis [43]. Significant enrichment was indicated by a minimum overlap of 3, p-value cut-off of 0.05, and minimum enrichment of 1.5.

### 4.8 Protein-protein interaction (PPI) network

To identify the potential interactions among the upregulated DEGs-encoded proteins, which were of particular interest compared to the downregulated ones, the Metascape database was also employed to construct the PPI network, which was then visualized by Cytoscape software (version 3.9.1). The connection degree of each protein, namely the number of proteins it connects, was calculated to evaluate its importance in this network. Significant PPI network modules were then identified via the Cytoscape plugin Molecular Complex Detection (MCODE) (version 2.0.2) with selection criteria: MCODE scores >5, degree cut-off = 2, node score cut-off = 0.2, maximum depth = 100 and k-score = 2.

### 4.9 Regulatory network of mRNAs and miRNAs

Targetscan (version 5.0) (https://www.targetscan.org/) and miRanda (version 3.3a) (http://www.microrna.org/microrna/) were used to predict potential target genes of DEmiRNAs, the strength of predicted miRNA-mRNA pairings met with both TargetScan_score ≥50 and miranda_Energy < −10 were screened out. Next, from predicted target mRNAs, only the ones belonging to overlapped DEGs (our dataset vs GSE100122) or hub DEGs of PPI modules were selected as the final candidate mRNAs, and their corresponding DEmiRNAs were matched. Only pairings with inverse expression, i.e., upregulated DEG and downregulated DEmiRNA or vice versa, were selected.

### 4.10 Quantitative real-time PCR (qRT-PCR)

**Fig. 5:**
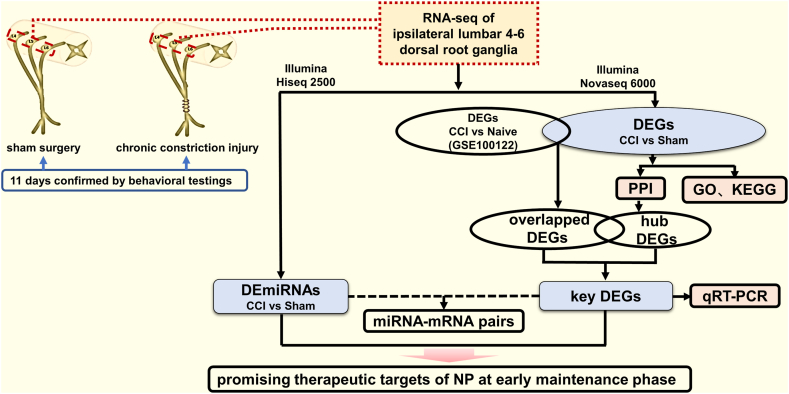
Schematic diagram of the study design. NP: neuropathic pain; CCI: chronic constriction injury; DEGs: differentially expressed mRNAs; DEmiRNAs: differentially expressed miRNAs; GO: Gene Ontology; KEGG: Kyoto Encyclopedia of Genes and Genomes; PPI: protein-protein interaction; qRT-PCR: quantitative real-time PCR.

Fifteen upregulated DEGs were selected for qRT-PCR validation. The concentration and quality of total RNA extracted from each sample (n = 7 per group) as described previously were determined with Epoch Microplate Spectrophotometer (Biotek, USA), and then reverse transcribed into first-strand cDNA using Eastep®RT Master Mix Kit (Promega, USA) following the manufacturer's instructions. Primer sequences are listed in Supplementary Table S1. The 20 μl PCR reaction mixture consisted of 10 μl 2 × ChemoHS SYBR Green qPCR Mix without ROX (Monad, China), 0.4 μl primers (10 nM, forward and reverse), 2 μl cDNA and nuclease-free water (up to 20 μl). Real‐time PCR reactions were performed on a Linegene 9600 Plus detection system (Bioer, Hangzhou, China) with thermal cycling program: incubation at 95 °C for 10 min, 40 cycles of denaturation at 95 °C for 10s, and annealing/elongation at 60 °C for 30s followed by melting curve analysis to confirm the specificity of the primers. Three biological replicates from each group and three technical replicates for each sample were set for analysis. Expression levels of these genes in the CCI-DRG compared with the sham-DRG were determined by the 2-ΔΔCt method. GAPDH was used as the internal reference. The overall study design has been depicted in a schematic diagram (Fig. 5).

### 4.11 Statistical analysis

The statistical analysis of behavioral testings and qRT-PCR were performed using GraphPad Prism 9 (GraphPad Software, La Jolla, CA, USA). Shapiro-Wilk test was used to test data normality. Behavioral testings were analyzed using two-way repeated measures ANOVA followed by Tukey's post-hoc test, and data were expressed as mean ± SD. Two-tailed unpaired *t*-test was used to analyze qRT-PCR results, and data were presented as mean ± SEM. p < 0.05 was considered statistically significant.

## 5 Conclusions

In conclusion, we have delineated transcriptomic signatures of rat ipsilateral DRG that underwent expression changes triggered by CCI, and gained a comprehensive landscape of the key mRNAs, miRNAs, and main pathophysiological processes involved in the persistence of NP at a relatively early maintenance phase. Candidate mRNAs and miRNAs, such as Atf3, Jun, Cd74, miR-21-5p, miR-34a-5p, miR-200a-3p, and macrophages-specific genes (Cx3cr1, Arg1, Cd68, Csf1r) may serve as promising therapeutic targets for NP. We will continue to focus on verifying the roles of these candidates by conducting preclinical and clinical experiments on both sexes in future studies.

## Author contributions

YQ, HW and LL designed, coordinated and supervised the study, and wrote the manuscript. YQ and QL carried out the experiments. YQ and RC collected and analyzed the data. All authors have read and approved the final submitted manuscript.

## Funding

This study was supported by the 11th Youth Development Fund of The 10.13039/501100017585First Hospital of Jilin University (No. JDYY11202030).

## Institutional review board statement

Not applicable.

## Informed consent statement

Not applicable.

## Data availability statement

RNA sequencing raw data generated in this study have been deposited in the Gene Expression Omnibus under the accession number GSE212311. Publicly available raw datasets GSE100122 provided by a previous study [9] was used in this work.

## Declaration of competing interest

The authors declare that they have no known competing financial interests or personal relationships that could have appeared to influence the work reported in this paper.
