# Supplementary material for: Neuroinflammation signatures in dorsal root ganglia following chronic constriction injury
Source: Heliyon. 2024 May 17;10(10):e31481. doi: 10.1016/j.heliyon.2024.e31481 (PMC11133895; doi:10.1016/j.heliyon.2024.e31481)

**Table S1.** Primer sequences for quantitative real-time PCR

|  | **Forward primer (5’-3’)** | **Reverse primer (5’-3’)** |
| --- | --- | --- |
| Cx3cr1 | CATGTGCAAGCTCACGACTG | GTTGCACTGTCCGGTTGTTC |
| Arg1 | CTGGGTGGAGACCACAGTATG | CCTTCAGGAGAAAGGCCACC |
| Cd68 | GACACTTCGGGCCATGCTTC | GGAGGACCAGGCCAATGATG |
| Csf1r | GGGAGAAGAGTAGGACCACC | AGTGCTCCTCTGACCCATGA |
| Gadd45a | CTGTGTGCTGGTGACGAACC | ACCCACTGATCCATGTAGCG |
| Adcyap1 | TAGCTTCGGCAAACAAGTCC | TACACATGGTCATTCGCGGC |
| Panx1 | GTGGCTGCACAAGTTCTTCC | AACTTCAGGTCGGAGCAGAG |
| Sox11 | CCGACGACCTCATGTTCGAC | GGTCCTTATCCACCAGCGAC |
| Ccl2 | AGCCAACTCTCACTGAAGCC | TGGGGCATTAACTGCATCTGG |
| Fcgr3a | TCCGTGGCAGTCTATGAGG | CAGATGGTGAGGTCGCAAG |
| Bdnf | TGGCAGGCTTTGATGAGACC | TCTCACCTGGTGGAACTCAG |
| Tyrobp | ACAGGCCCAGAGTGACAATTAC | CAATCCCAGCCAGTACACCC |
| Ctss | AGTCATTTCGACCAGCCTCC | GCACCCAAAACAGCCATCTC |
| Vtcn1 | GGCTTTGGTATTTCAGGCAAGC | GTCAGGTTCAAACGTGCAGC |
| Csrp3 | TGTAAGGTCTGCTATGGGCG | GCTGGCTTTGGGGATTGTTG |
| Gapdh | TCTCTGCTCCTCCCTGTTCT | GTTCACACCGACCTTCACCA |

**Figure S1.** MiRNA-mRNA regulatory network. (A) Selected key DEGs for constructing miRNA-mRNA network. 113 DEGs in yellow circle are overlapped genes between our dataset and GSE100122 dataset, 58 DEGs in blue circle are hub genes from 5 PPI modules, 16 genes are shared by these two sets of genes. (B) Highly predicted miRNA-mRNA pairs between 16 DEmiRNAs and selected key DEGs. Blue and red circle indicate downregulated and upregulated miRNAs respectively, red and blue square indicate upregulated and downregulated mRNAs respectively.

**
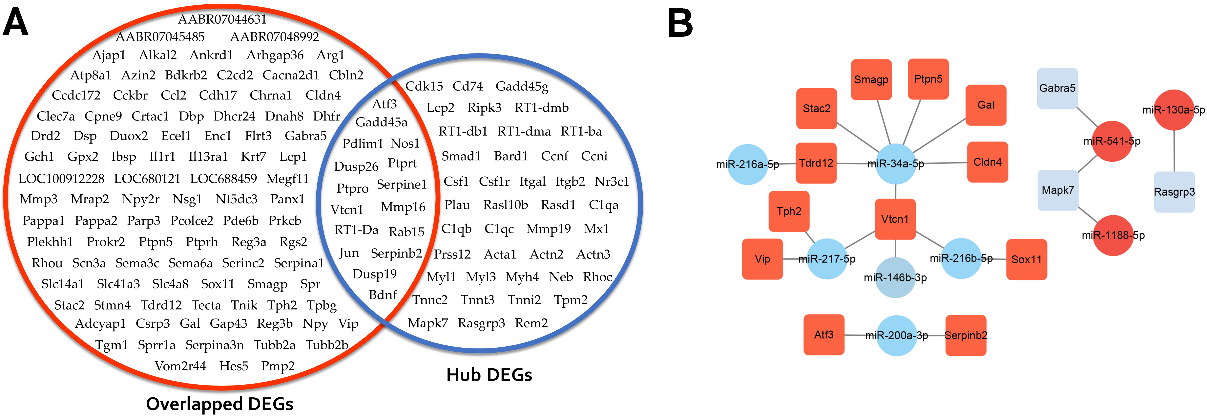
**

**Figure S2.** qRT-PCR validation for expressions of selected DEGs from RNA-seq analysis. Values are expressed as mean ± SEM. *p <0.05, **p <0.01, ***p <0.001, ****p <0.0001 CCI group (n=3) compared with the sham group (n=3).


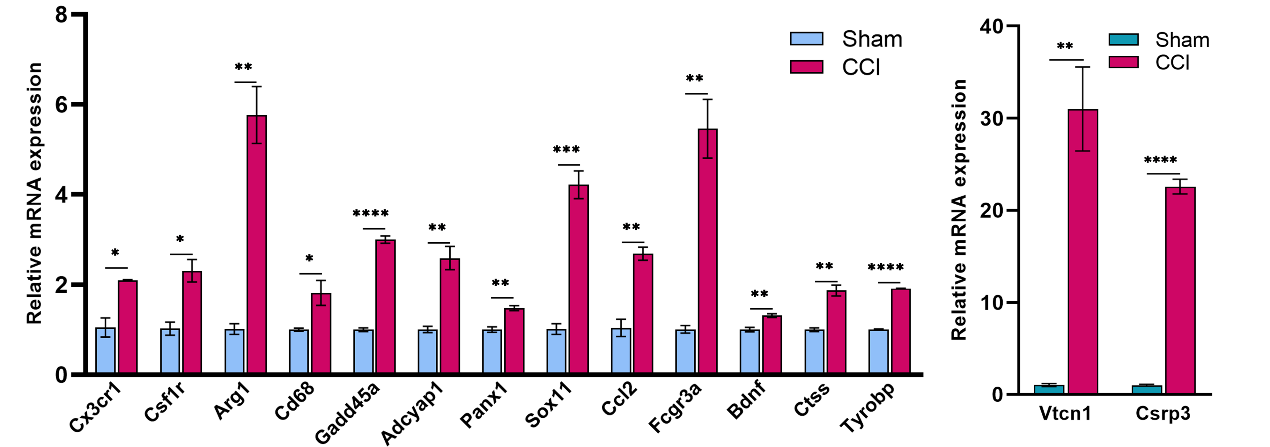

Supplement: Multimedia component 1 [file mmc1.docx]
